# Bionic Technology in Prosthetics: Multi-Objective Optimization of a Bioinspired Shoulder-Elbow Prosthesis with Embedded Actuation

**DOI:** 10.3390/biomimetics11010079

**Published:** 2026-01-19

**Authors:** Jingxu Jiang, Gengbiao Chen, Xin Wang, Hongwei Yan

**Affiliations:** 1 International College of Engineering, Changsha University of Science and Technology, Changsha 410114, China; jiangjingxu@csust.edu.cn; 2 College of Mechanical and Vehicle Engineering, Changsha University of Science and Technology, Changsha 410114, China; 23203030740@csust.edu.cn (X.W.); 21203030682@csust.edu.cn (H.Y.)

**Keywords:** bionic technology, upper-limb prosthesis, bioinspired design, spherical parallel mechanism (SPM), shape memory alloy (SMA), multi-objective optimization, embedded actuation, integrated prosthetic system

## Abstract

The development of upper-limb prostheses is often hindered by limited dexterity, a restricted workspace, and bulky designs, primarily due to performance limitations in proximal joints like the shoulder and elbow, which contribute to high user abandonment rates. To overcome these challenges, this paper presents a novel, bioinspired, and integrated prosthetic system as an advancement in bionic technology. The design incorporates a shoulder joint based on an asymmetric 3-RRR spherical parallel mechanism (SPM) with actuators embedded within the moving platform, and an elbow joint actuated by low-voltage Shape Memory Alloy (SMA) springs. The inverse kinematics of the shoulder mechanism was established, revealing the existence of up to eight configurations. We employed Multi-Objective Particle Swarm Optimization (MOPSO) to simultaneously maximize workspace coverage, enhance dexterity, and minimize joint torque. The optimized design achieves remarkable performance: (1) 85% coverage of the natural shoulder’s workspace; (2) a maximum von Mises stress of merely 3.4 MPa under a 40 N load, ensuring structural integrity; and (3) a sub-0.2 s response time for the SMA-driven elbow under low-voltage conditions (6 V) at a motion velocity of 6°/s. Both motion simulation and prototype testing validated smooth and anthropomorphic motion trajectories. This work provides a comprehensive framework for developing lightweight, high-performance prosthetic limbs, establishing a solid foundation for next-generation wearable robotics and bionic devices. Future research will focus on the integration of neural interfaces for intuitive control.

## 1. Introduction

Upper-limb loss profoundly impacts an individual’s ability to perform daily activities, work, and engage in social life [1,2]. The restoration of motor function through prosthetic devices remains a paramount challenge in rehabilitation engineering and robotics. While current commercial myoelectric prostheses can restore basic grasping functions, they often fall short of user expectations, leading to high abandonment rates, estimated to be between 23% and 44% [3]. The core of this issue frequently lies in the inadequate performance of the proximal joints, particularly the shoulder and elbow, which form the crucial foundation for positioning the terminal device in space [4].

Traditional shoulder prostheses, often based on serial mechanisms, are plagued by several inherent limitations. Their sequential architecture, while providing a large workspace, places actuators distally, resulting in high inertial forces, significant reflected mass, and elevated power consumption [5,6]. This often leads to delayed response, unnatural motion, and user fatigue [7]. Furthermore, the workspace of conventional symmetric shoulder mechanisms covers less than 60% of the natural human shoulder’s asymmetric range of motion (e.g., flexion/extension: −90° to 20°; abduction/adduction: −90° to 10°) [8,9], severely limiting functional reach. Although parallel mechanisms, particularly spherical parallel mechanisms (SPMs), offer a promising alternative due to their compact topology, high stiffness, and payload-to-weight ratio [10,11], their design often involves a trade-off between workspace size, dexterity, and structural interference [12,13]. A significant drawback of many existing designs is the placement of actuators away from the joint center, increasing the system’s inertia and volume [14].

At the elbow, the actuation challenge revolves around achieving a balance between force, speed, weight, and efficiency. Conventional motor-driven systems, often coupled with harmonic drives or gears, can provide substantial torque but are typically heavy, noisy, and energetically inefficient [15,16]. Shape Memory Alloys (SMAs), known for their high power density and silent operation, present a compelling alternative for anthropomorphic actuation [17,18]. However, their application in prosthetics has been hindered by challenges such as slow response times due to inefficient thermal management, limited cycle life, and control complexities arising from their nonlinear hysteresis [19,20]. While control strategies based on surface electromyography (sEMG) have advanced [21,22], the actuation hardware often lacks the responsiveness and biomimetic fidelity needed for seamless integration into intuitive control loops.

Beyond the mechanics of individual joints, the overall performance of a prosthetic arm is dictated by the synergistic coordination of its constituent joints. This necessitates a system-level optimization approach that simultaneously addresses multiple, often competing, objectives: maximizing workspace coverage to match the human anatomical range [23,24], enhancing kinematic dexterity for agile and omnidirectional movement [25,26], and minimizing joint torques to enable the use of smaller, lighter actuators and reduce energy consumption [27,28]. Traditional design methods, which often optimize for a single objective or use sequential optimization routines, fail to capture these complex trade-offs, leading to suboptimal system-level performance [29,30]. Multi-objective optimization algorithms, such as Multi-Objective Particle Swarm Optimization (MOPSO) [31,32] and Non-dominated Sorting Genetic Algorithm (NSGA-II) [33], have been employed in robotic design to find Pareto-optimal solutions that balance these constraints. However, their application to the integrated, biomimetic design of a complete shoulder-elbow prosthetic system remains underexplored.

In summary, the field of upper-limb prosthetics grapples with a tripartite challenge: (1) achieving shoulder mechanics that are simultaneously compact, dexterous, and powerful; (2) developing elbow actuation that is efficient, responsive, and quiet; and (3) implementing a holistic design strategy that co-optimizes these subsystems for superior global performance. Most existing research addresses these challenges in isolation [34,35]. A synergistic approach that tackles them concurrently is essential for a paradigm shift towards prostheses that are not just functional tools, but truly natural extensions of the human body.

This study addresses this critical gap by introducing a novel, bioinspired, integrated upper-limb prosthesis that jointly tackles the challenges of shoulder and elbow function. The key contributions of this work are:

(1) A structurally innovative shoulder joint design: We propose an improved asymmetric 3-RRR Spherical Parallel Mechanism (SPM) with actuators embedded directly within the moving platform. This groundbreaking configuration shortens the power transmission path. Compared to our team’s earlier serial-link prototype, this design achieves a 32% reduction in inertia and overall mass, and enhances integration, leading to more natural and responsive anthropomorphic motion.

(2) A novel efficient elbow actuation strategy: We develop a dual-fork arm architecture driven by low-voltage Shape Memory Alloy (SMA) springs. This design enables efficient and stable actuation under low-voltage conditions (6 V) for flexion-extension, with passive return upon cooling, offering a high power-density and silent alternative to traditional bulky motors.

(3) A comprehensive multi-objective optimization framework: We establish a kinematic model using reciprocal screw theory and the closed-loop vector method and employ the MOPSO algorithm to co-optimize workspace coverage, motion dexterity (using the condition number of the Jacobian), and joint torque. This integrated framework improved the convergence efficiency towards Pareto-optimal solutions by 40%, effectively balancing these competing design objectives.

(4) Experimental validation: The optimized design is rigorously validated through finite element analysis (FEA) under load, kinematic simulations demonstrating smooth motion trajectories, and the successful testing of a modular physical prototype, confirming its practical feasibility and performance.

The remainder of this paper is organized as follows: **Section 2** details the mechanical design, kinematic modeling, and multi-objective optimization of the proposed shoulder-elbow prosthesis. **Section 3** provides the simulation and experimental validation results. **Section 4** discusses the findings, limitations, and future work. Finally, **Section 5** concludes the paper. 

## 2. Materials and Methods

### 2.1. Integrated Biomimetic Mechanical Design

The human arm is a complex kinematic system comprising multiple bones and muscles, including the shoulder, elbow, and wrist joints. The coordinated motion of these joints enables a wide range of compound movements and functional tasks. To replicate the physiological characteristics of the human shoulder, a spherical parallel mechanism offers a highly anthropomorphic solution.

#### 2.1.1. Design Philosophy and Overall Architecture

**Figure 1:**
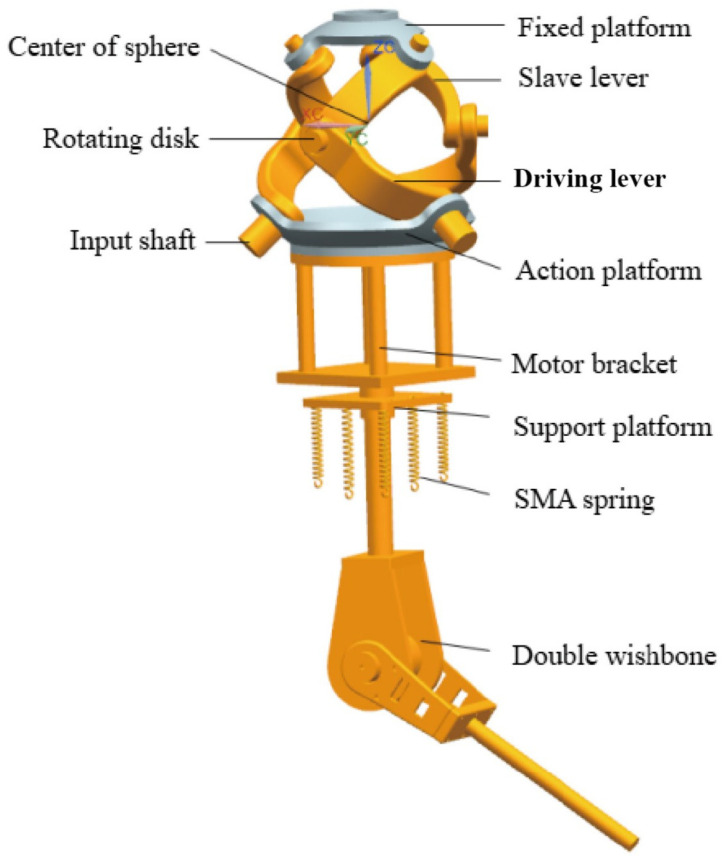
Overall structure of the upper-limb prosthesis.

The prosthetic system was conceived with integration and biomimesis as its core principles. The overall architecture, as shown in Figure 1, illustrates the cohesive assembly of the shoulder and elbow modules. The fixed platform of the shoulder joint serves as the stable foundation, connected to the upper arm housing, which contains the actuation systems. The elbow joint is distally connected, culminating in the forearm segment. This modular, split-type design emphasizes practical prosthetic requirements, significantly reducing mass and improving portability and maintainability.

#### 2.1.2. Shoulder Joint: Innovative Design with Embedded Actuation

**Figure 2:**
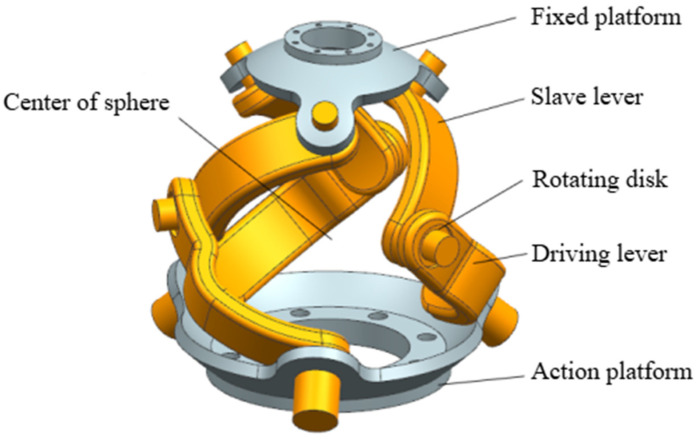
Shoulder joint structure.

The shoulder joint’s design is a cornerstone of this work. Figure 2 provides a detailed view of the compact 3-RRR SPM structure with actuators embedded within the moving platform. The image clearly shows the three kinematic chains, each consisting of an active link and a passive link, connecting the fixed base to the moving platform. All rotational joint axes are designed to intersect at a common center, enabling pure rotational motion. This configuration is structurally analogous to the human shoulder joint, providing a highly anthropomorphic solution.

#### 2.1.3. Elbow Joint: Efficient Actuation Strategy with SMA Springs

**Figure 3:**
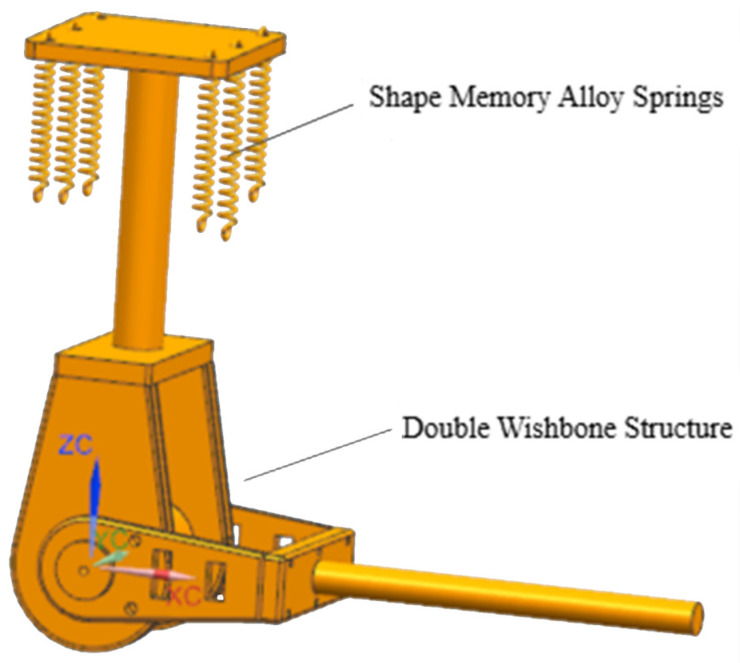
Elbow joint structure.

The elbow joint employs a novel actuation strategy. Figure 3 depicts the dual-fork arm architecture driven by low-voltage Shape Memory Alloy (SMA) springs. The diagram shows the central rotating shaft supported by tapered roller bearings, ensuring stable rotation. The three symmetrically arranged sets of SMA springs are visible, connected via cables to apply torque to the shaft. The end caps providing axial fixation are also illustrated, highlighting the mechanical design that enables efficient flexion and passive return upon cooling.

### 2.2. Kinematic Modeling of the Shoulder Joint

#### 2.2.1. Degree-of-Freedom Analysis

**Figure 4:**
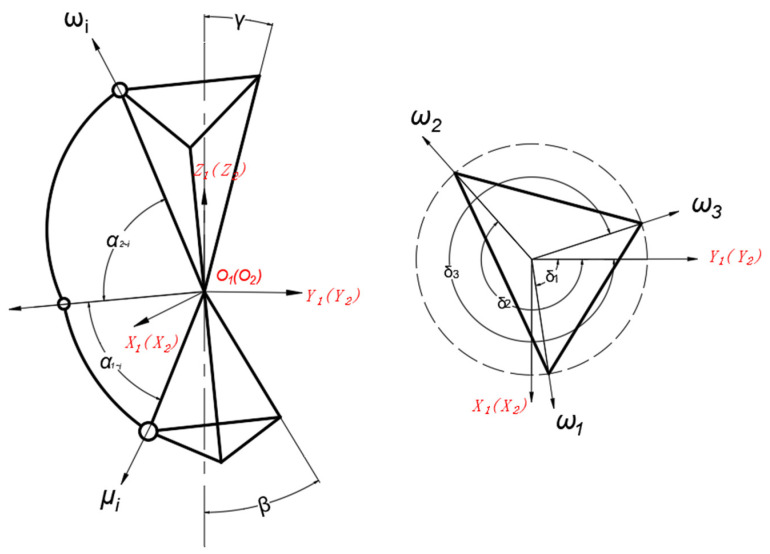
Schematic of the 3-DOF spherical parallel mechanism.

The 3-RRR SPM is a 3-DOF spherical structure consisting of three rotational joints. It connects a stationary fixed platform to a movable platform through parallel kinematic chains. As shown in Figure 4, a fixed coordinate system *O*_1_-*X*_1_*Y*_1_*Z*_1_ is established on the fixed platform, and a moving coordinate system *O*_2_-*X*_2_*Y*_2_*Z*_2_ is defined on the moving platform. The origins of both coordinate systems coincide with the rotation center of the spherical joint, simplifying motion analysis.

The three axes of the fixed coordinate system correspond to the principal anatomical motion planes of the human body: the X-axis aligns with the coronal plane, the Y-axis with the sagittal plane, and the Z-axis is perpendicular to both, representing the horizontal rotation axis. This setup facilitates direct mapping and analysis of coordinated motion with the human shoulder joint.

For each kinematic chain of the 3-RRR mechanism:

μ_i_ denotes the unit vector from the moving platform to the first rotational joint (active link);

ω_i_ denotes the unit vector from the active link to the second rotational joint (passive link);

ν_i_ denotes the unit vector from the passive link to the connecting axis of the fixed platform.

These vectors are defined within their respective kinematic chains and collectively determine the spatial motion characteristics of the mechanism. The connection points of these links form an irregular triangular arrangement on both platforms, ensuring a balanced trade-off between structural rigidity and flexibility.

The “initial posture” is defined as the configuration where the projections of all vectors μ_i_ and ω_i_ onto the *X*_1_*Y*_1_ plane coincide. In this state, there is no relative motion between the platforms, and the moving and fixed coordinate systems are identical. This configuration serves as the reference for zero-position analysis.

The degree-of-freedom analysis utilizes reciprocal screw theory. By computing the common constraint screw between the kinematic chains, this method enables an accurate evaluation of the mechanism’s mobility. The motion screw system of the first kinematic chain, based on the fixed coordinate system *O*_1_-*X*_1_*Y*_1_*Z*_1_, is given by:

(1) $$\begin{array}{l} {\$}_{11}=\left(\begin{array}{ccccc} a_{11} & b_{11} & c_{11};0 & 0 & 0 \end{array}\right)\\ {\$}_{12}=\left(\begin{array}{ccccc} a_{12} & b_{12} & c_{12};0 & 0 & 0 \end{array}\right)\\ {\$}_{13}=\left(\begin{array}{ccccc} a_{13} & b_{13} & c_{13};0 & 0 & 0 \end{array}\right) \end{array}$$

Table 1 presents the key parameters of the 3-RRR SPM and their physical significance, forming the basis for subsequent modeling and simulation.

The reciprocal screw system is expressed as:

(2) $$\begin{array}{c} {{\$}^{r}}_{11}=\left(\begin{array}{ccccc} 1 & 0 & 0;0 & 0 & 0 \end{array}\right)\\ {{\$}^{r}}_{12}=\left(\begin{array}{ccccc} 0 & 1 & 0;0 & 0 & 0 \end{array}\right)\\ {{\$}^{r}}_{13}=\left(\begin{array}{ccccc} 0 & 0 & 1;0 & 0 & 0 \end{array}\right) \end{array}$$

In the fixed coordinate system, the motion screw system of the i-th kinematic chain can be represented by three reciprocal screws, each constraining the motion of the moving platform along the *X*_1_, *Y*_1_, and *Z*_1_ axes. This implies that each chain individually constrains the platform in all three coordinate directions. Since the components of these constraint forces along the coordinate axes are linearly dependent, they cannot independently restrict the platform’s motion, reducing the number of effective constraints. Ultimately, this combination yields three independent common constraints (*λ* = 3).

The degrees of freedom (DOF) of the shoulder joint mechanism can be determined using the following formula:

(3) $$M=d(l-n-1)+\sum_{i=1}^{n}f_{i}$$

Here, d = 6 − λ represents the mobility (or order) of the mechanism, l is the number of components, n is the total number of kinematic pairs (joints), and *f_i_* denotes the degree of freedom of the i-th kinematic pair. Substituting the known values (d = 3, l = 8, n = 9, and all joints are single-DOF revolute pairs), the DOF of the shoulder joint mechanism is calculated to be three.

#### 2.2.2. Inverse Kinematics

The inverse kinematics problem involves determining the input joint angles θ_i_ (i = 1, 2, 3) from the orientation parameters of the moving platform, denoted as *m*_a_, *m*_b_ and *m*_c_. The analysis is performed using the closed-loop vector method, suitable for modeling the three rotational kinematic chains.

The central task is to define the unit vectors *ω_i_*, *ν_i_* and *µ_i_*, which define the orientations of the rotational axes relative to the moving platform coordinate system. The posture of the moving platform is expressed using *x*-*y*-*z* Euler angles:

(4) $$\begin{array}{c} Q_{xy}(m_{a},m_{b},m_{c})=R_{x}(m_{a})R_{y}(m_{b})R_{z}(m_{c})\\ =\left[\begin{array}{ccc} 1 & 0 & 0\\ 0 & \cos m_{a} & -\sin m_{a}\\ 0 & \sin m_{a} & \cos m_{a} \end{array}\right]\left[\begin{array}{ccc} \cos m_{b} & 0 & \sin m_{b}\\ 0 & 1 & 0\\ -\sin m_{b} & 0 & \cos m_{b} \end{array}\right]\left[\begin{array}{ccc} \cos m_{c} & -\sin m_{c} & 0\\ \sin m_{c} & \cos m_{c} & 0\\ 0 & 0 & 1 \end{array}\right] \end{array}$$

The rotation transformation matrix *Q* represents the orientation of the moving coordinate system relative to the fixed system. The inverse matrix *Q*^−1^ expresses the rotation from the fixed to the moving coordinate system when the latter is used as the reference frame.

The unit vectors of the rotational axes—ω_i_^1^, ν_i_^1^ and µ_i_^1^—are defined in the moving coordinate system. Their relationships are described as follows:

(5) $$\begin{array}{l} \omega_{i}^{1}=Q^{-1}\omega_{i}\\ v_{i}^{1}=Q^{-1}v_{i}\\ \mu_{i}^{1}=Q^{-1}\mu_{i} \end{array}$$

The unit vector µ_i_^1^ is expressed as:

(6) $$\mu_{i}^{1}=R_{z}(\delta_{i})R_{x}(\frac{\pi }{2}-\beta ){\left[0,1,0\right]}^{T}\;(i=1,2,3)$$

where R_a_(b) denotes a rotation about axis a by angle b. Rotating the active link about µ_i_^1^ by angle θ_i_ yields the vector ν_i_^1^:

(7) $$v_{i}^{1}=R_{z}(\delta_{i})R_{x}(\frac{\pi }{2}-\beta )R_{y}(\theta_{i})R_{z}(-\alpha_{1\sim i}){\left[0,1,0\right]}^{T}\;(i=1,2,3)$$

The unit vector ω_i_1, representing the axis of the revolute joint connected to the fixed platform, is derived from the orientation of the fixed platform as observed in the moving coordinate system:

(8) $$\omega_{i}^{1}=Q^{-1}\omega_{i}^{\prime }$$

In the initial posture, the unit vector ω_i_1 defines the rotation axis of the revolute joint connected to the fixed platform:

(9) $$\omega_{i}^{\prime }=R_{z}(\delta_{i})R_{x}(\gamma -\frac{\pi }{2}){\left[0,1,0\right]}^{T}\;(i=1,2,3)$$

The unit vectors must satisfy the closed-chain constraint equations:

(10) $$v_{i}^{1}\omega_{i}^{1}=\cos(\alpha_{2\sim i})\;(i=1,2,3)$$

Substituting Equations (4)–(9) into Equation (10) yields the expression for y_i_:

(11) $$M_{ai}y_{i}^{2}+M_{bi}y_{i}+M_{ci}=0$$

(12) $$y_{i}=\tan(\frac{\theta_{i}}{2}),\sin\theta_{i}=\frac{2y_{i}}{1+y_{i}^{2}},\cos\theta_{i}=\frac{1-y_{i}^{2}}{1+y_{i}^{2}}(i=1,2,3)$$

where

$$\begin{array}{l} \begin{array}{l} M_{ai}=\omega_{ix}(-b_{1}+b_{3})+\omega_{i_{y}}(b_{4}-b_{5})+\omega_{i_{z}}(b_{7})-\cos(\alpha_{2\sim i})\\ M_{bi}=\omega_{ix}(2b_{2})+\omega_{i_{y}}(2b_{6})+\omega_{i_{z}}(2b_{8})\\ M_{ci}=\omega_{ix}(b_{1}+b_{3})+\omega_{i_{y}}(b_{4}+b_{5})+\omega_{i_{z}}(b_{7})-\cos(\alpha_{2\sim i}) \end{array}\\ \begin{array}{l} b_{1}=\sin(\alpha_{1\sim i})\cos(\delta_{i})\\ b_{2}=-\sin(\alpha_{1\sim i})\sin(\delta_{i})\cos(\beta )\\ b_{3}=-\cos(\alpha_{1_{\sim }i})\sin(\delta_{i})\sin(\beta )\\ b_{4}=\cos(\alpha_{1\sim i})\cos(\delta_{i})\sin(\beta )\\ b_{5}=\sin(\alpha_{1\sim i})\sin(\delta_{i})\\ b_{6}=\sin(\alpha_{1\sim i})\cos(\delta_{i})\cos(\beta )\\ b_{7}=\cos(\alpha_{1\sim i})\cos(\beta )\\ b_{8}=-\sin(\alpha_{1\sim i})\sin(\beta ) \end{array} \end{array}$$

M_ai_, M_bi_ and M_ci_ are functions relating the mechanism’s parameters to the posture angles m_a_, m_b_ and m_c_. Solving Equation (11) gives the input angle of the active link:

(13) $$\theta_{i}=2\mathrm{Atan}2(-M_{yi}\pm \sqrt{M_{yi}^{2}-4M_{xi}M_{zi}},2M_{xi})(i=1,2,3)$$

where atan2 denotes the two-argument arctangent function.

**Figure 5:**
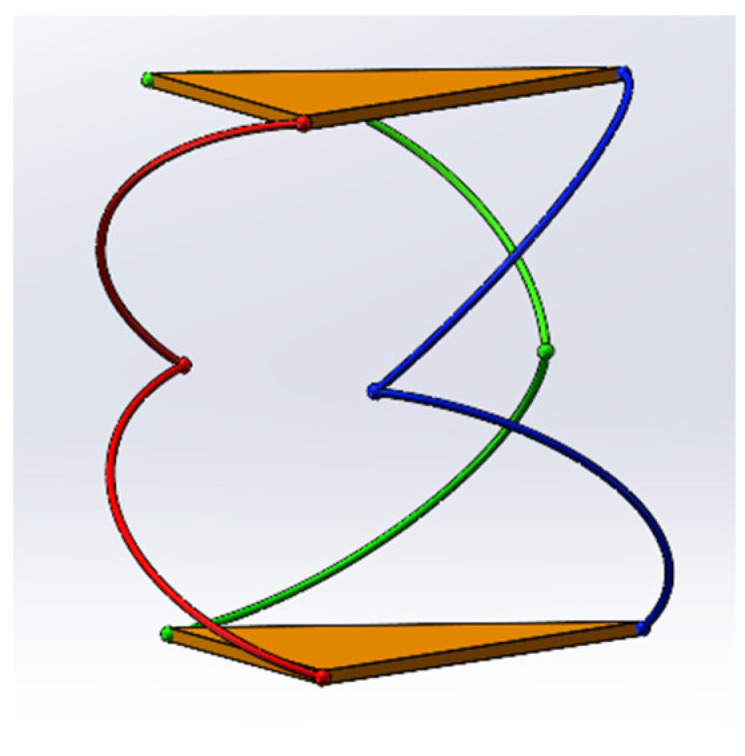
Example of a valid inverse kinematic configuration.

Equation (13) indicates that each kinematic chain admits two possible inverse kinematic solutions, corresponding to forward or reverse rotations. With three chains, the total number of potential solutions is 2^3^ = 8. However, only a subset corresponds to valid, feasible configurations for achieving the desired moving platform orientation, as illustrated in Figure 5.

#### 2.2.3. Forward Kinematics

The forward kinematics problem involves determining the posture angles ma, mb and mc from the input angles η_i_(i = 1,2,3) of the active links. This problem is generally more complex and nonlinear than inverse kinematics and may have multiple valid solutions.

The unit vectors ν_i_^1^ and µ_i_^1^ are defined as in Equations (6) and (7). For ω_1_^1^, the geometric constraints of the fixed platform must be considered:

(14) $$\omega_{1}^{1}=R_{z}(\delta_{i})R_{x}(\frac{\pi }{2}-\beta )R_{y}(\theta_{i})R_{z}(-\alpha_{1\sim i})R_{y}(\kappa_{1})R_{z}(-\alpha_{1\sim 1}){\left[0,1,0\right]}^{T}$$

Here, K_1_ is an unknown variable representing the rotation angle of the intermediate joint in the first chain. The unit vector ω_2_^1^ is derived based on ω_1_^1^:

(15) $$\omega_{2}^{1}=R_{z}(\delta_{i})R_{x}(\frac{\pi }{2}-\beta )R_{y}(\theta_{i})R_{z}(-\alpha_{1\sim i})R_{y}(\kappa_{1})R_{z}(-\alpha_{1\sim 1})R_{y}(\eta_{1})R_{z}(\tau_{12}){\left[0,1,0\right]}^{T}$$

The variable τ_1_^2^ denotes the angle between ω_1_^1^ and ω_2_^1^, and ϑ_1_ is another unknown variable representing the rotation about ω_1_^1^. The unit vector for the third chain, ω_3_^1^ is derived from ω_2_^1^:

(16) $$\omega_{3}^{1}=R_{z}(\delta_{i})R_{x}(\frac{\pi }{2}-\beta )R_{y}(\theta_{i})R_{z}(-\alpha_{1\sim i})R_{y}(\kappa_{1})R_{z}(-\alpha_{1\sim 1})R_{y}(\vartheta_{1}-\xi_{213})R_{z}(\tau_{13}){\left[0,1,0\right]}^{T}$$

Here, ξ_123_ is the dihedral angle between the plane defined by ω_1_^1^ and ω_2_^1^ and the plane defined by ω_3_^1^; it is a fixed structural parameter.

According to the closed-loop constraints:

(17) $$v_{i}^{1}\omega_{i}^{1}=\cos(\alpha_{1\sim i})\;(i=2,3)$$

Substituting the relevant equations into Equation (17) yields a system with unknown variable *K*_1_:

(18) $$A_{j}\sin\kappa_{1}+B_{j}\cos\kappa_{1}+C_{j}=0(j=1,2)$$

where *A*_j_, *B*_j_ and *C*_j_ are functions of the geometric parameters, input angles *η*_i_, and unknown variable ϑ_1_. Solving this system gives *K*_1_:

(19) $$\left\{\begin{array}{l} \sin\kappa_{1}=\frac{B_{1}C_{2}-B_{2}C_{1}}{A_{1}B_{2}-A_{2}B_{1}}\\ \cos\kappa_{1}=\frac{A_{2}C_{1}-A_{1}C_{2}}{A_{1}B_{2}-A_{2}B_{1}} \end{array}\right.$$

Using the trigonometric identity sin^2^*K*_1_ + cos^2^*K*_1_ = 1, and letting x = tanϑ1/2 and using the substitutions sinϑ1 = 2x/(1 + x^2^), cosϑ1 = (1 − x^2^)/(1 + x^2^), Equation (19) simplifies to:

(20) $$\sum_{i=0}^{8}c_{i}x^{i}=0$$

This is an eighth-degree polynomial in *x*, where the coefficients *c_i_* are functions of the structural parameters and input angles. Solving this polynomial provides the analytical solution for *x*, and thus:

(21) $$\vartheta_{1}=2\arctan x$$

Substituting this value into Equation (19) yields *K*_1_, and subsequently, the unit vectors ω_i_^1^ (i = 1, 2, 3) can be computed from Equations (14)–(16). Then, based on Equation (8):

(22) $$Q=\left[\omega_{1}^{\prime }\;\omega_{2}^{\prime }\;\omega_{3}^{\prime }\right]{\left[\omega_{1}^{1}\;\omega_{2}^{1}\;\omega_{3}^{1}\right]}^{-1}$$

Finally, the *x*-*y*-*z* Euler angles are determined by solving the inverse of the rotation matrix:

(23) $$Q_{xyz}(m_{a},m_{b},m_{c})=R_{x}(m_{x})R_{y}(m_{y})R_{z}(m_{z})=\left[\begin{array}{ccc} r_{11} & r_{12} & r_{13}\\ r_{21} & r_{22} & r_{23}\\ r_{31} & r_{32} & r_{33} \end{array}\right]$$

Thus:

(24) $$\left\{\begin{array}{l} m_{a}=A\tan2(-r_{23},r_{33})\\ m_{b}=A\tan2(r_{13},\sqrt{r_{11}^{2}+r_{12}^{2}})\\ m_{c}=A\tan2(-r_{12},r_{11}) \end{array}\right.$$

The number of forward kinematic solutions is constrained by the number of real roots of the eighth-degree polynomial. While up to eight solutions are theoretically possible, only those that maintain motion continuity and physical feasibility are valid. The system must filter solutions based on the “solution trajectory continuity principle,” retaining only physically feasible and continuous motion paths. Figure 5 illustrates a valid configuration.

### 2.3. Multi-Objective Optimization Framework: Analysis and Algorithm Design

The design of the biomimetic shoulder joint must balance multiple performance metrics:

(1) Anthropomorphic appearance for better coordination with the human body.
(2) Motion reproduction capability, requiring an extensive workspace.
(3) Motion flexibility to perform high-frequency, complex movements.
(4) Lightweight design to reduce user burden, achieved by selecting small motors to minimize driving torque.

The multi-objective particle swarm optimization (MOPSO) algorithm was employed to handle these conflicting objectives simultaneously, seeking a set of Pareto-optimal solutions. The three optimization objectives were: maximizing the workspace, enhancing motion dexterity, and minimizing joint torque.

#### 2.3.1. Dexterity Analysis of the Shoulder Joint Mechanism

For a 3-DOF parallel mechanism with constant link lengths, only angular velocity is transmitted. A deviation in the input angular velocity ω causes a corresponding deviation in the output angular velocity (the rate of change of ϑ):

According to norm theory:

(25) $$\omega +△\omega =J(\dot{\vartheta }+△\dot{\vartheta })$$

(26) $$\begin{array}{l} ∥△\omega ∥=∥J△\dot{\vartheta }∥\leq ∥J∥\cdot ∥△\dot{\vartheta }∥\\ ∥\dot{\vartheta }∥=∥J^{-1}△\omega ∥\leq ∥J^{-1}∥\cdot ∥△\omega ∥ \end{array}$$

where ‖·‖ denotes the matrix norm. The relative deviation in angular velocity is:

(27) $$\frac{∥△\omega ∥}{∥\omega ∥}\leq ∥J∥\cdot ∥J^{-1}∥\frac{∥△\dot{\vartheta }∥}{∥\dot{\vartheta }∥}$$

Let k(*J*) = ‖J‖·‖J^−1^‖ be the condition number of the Jacobian matrix, measuring mechanism dexterity. Using the spectral norm:

(28) $$∥J∥={\left[\max\lambda (J^{T}J)\right]}^{\frac{1}{2}}$$

where maxλ(J^T^J) is the largest eigenvalue of J^T^J. If J^T^J is nonsingular and positive definite, all its eigenvalues are positive. The largest singular value of *J* is the square root of maxλ(J^T^J), thus:

(29) $$∥J∥=\sigma_{\max}(J)$$

Combining Equations (28)–(30), the dexterity measure is:

(30) $$k(J)==\frac{\sigma_{\max}(J)}{\sigma_{\min}(J)}$$

where σ_max_(*J*) and σ_min_(*J*) are the largest and smallest singular values of *J*, respectively. A large σ_max_(*J*) implies that a small input velocity deviation can cause a significant output deviation, indicating poor control accuracy and a higher risk of singularities, thus reducing dexterity. A small σ_min_(*J*) indicates more precise end-effector control and higher dexterity. When σ_max_(*J*) and σ_min_(*J*) are close (ideally, both equal to 1), the mechanism achieves optimal dexterity.

#### 2.3.2. Force Jacobian Matrix Analysis of the Shoulder Joint

Assuming all components are rigid bodies and neglecting non-ideal factors like joint clearance and friction, let *τ* be the driving torque vector of each branch, and M be the external torque vector applied to the moving platform. Based on the principle of virtual work and the velocity Jacobian matrix, the relationship between external torque M and joint torque τ is:

(31) $$\tau =J^{T}M$$

Equation (31) defines the force Jacobian matrix *J^T^*, which maps the external torque *M* to the required joint torques *τ*. If the total gravitational load of the human upper limb is *G* and the distance from its line of action to the shoulder joint center is *r_G_*, the equivalent external torque is:

(32) $$\tau =J^{T}({\left[\begin{array}{ccc} 0 & 0 & G \end{array}\right]}^{T}\times {\left[\begin{array}{ccc} 0 & 0 & r_{G} \end{array}\right]}^{T})$$

#### 2.3.3. Workspace Analysis of the Shoulder Joint

The workspace of a parallel mechanism is determined by geometric dimensions, joint limits, and link interference. The 3-RRR SPM has a smaller and more complex workspace than serial mechanisms. Workspace analysis involves:

(1) Positional workspace analysis: The range of positions achievable for a given orientation.
(2) Orientational workspace analysis: The range of orientations achievable for a given position.

For the 3-RRR SPM, analysis must consider the existence of kinematic solutions and potential interference. Since active and passive links lie on different spherical surfaces, mutual interference between them does not occur.

Interference Between Active Links and the Moving Platform: This is related to the input angular displacement *φ_zi_* (i = 1, 2, 3). Constraining *φ_zi_* within [*φ_zi_* _min_, *φ_zi max_*] avoids interference.

Interference Between Passive Links and the Fixed Platform: This depends on the angular displacement *φ_ci_*, which must be limited within [φ_ci min_, φ_ci max_].

Interference Between Adjacent Links of the Same Type: To prevent this, the shortest distance between the central axes of adjacent branches must exceed the link cross-sectional width *b*. The condition is:

(33) $$\begin{array}{l} D_{ij}^{e}\geq b(i,j=1,2;1,3;2,3)\\ D_{ij}^{f}\geq b(i,j=1,2;1,3;2,3)\\ D_{ij}^{e}=r_{1}\frac{v_{j}\cdot (v_{i}\times u_{i})}{\left\|\left.v_{i}\times u_{i}\right\|\right.}\\ D_{ij}^{f}=r_{2}\frac{v_{j}\cdot (w_{i}\times v_{i})}{\left\|\left.w_{i}\times v_{i}\right\|\right.} \end{array}$$

where *b* is the link width, *D^i^_ij_* is the shortest distance between the *i*-th active branch’s revolute joint and the *j*-th branch’s plane, *D^f^_ij_* is the shortest distance between the *i*-th and *j*-th passive branches, and *r*_1_ and *r*_2_ are the radii of the active and passive links, respectively.

The theoretical workspace is determined solely by kinematic solutions, ignoring physical constraints. The actual workspace incorporates physical limitations like interference, resulting in a smaller usable range.

**Figure 6:**
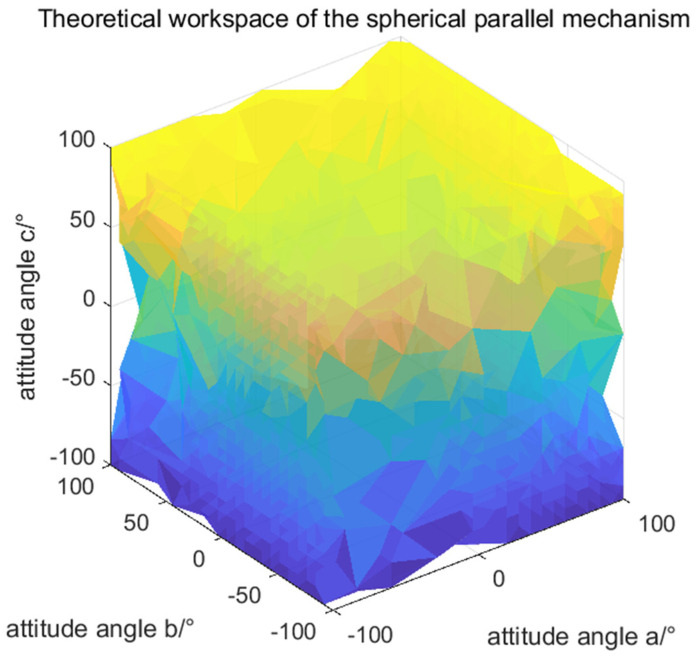
Theoretical workspace of the spherical parallel mechanism.

For the 3-RRR SPM, the moving platform undergoes pure rotation, so its workspace is defined by three orientation angles: *m_a_*, *m_b_* and *m_c_*. The theoretical workspace was computed via discretization, sampling over −180° ≤ *m_a_* ≤ 180°, −180° ≤ *m_b_* ≤180°, −180° ≤ *m_c_* ≤ 180°. For each sampled point, the inverse kinematics were solved to check for a valid solution. The resulting distribution is shown in Figure 6.

The human shoulder’s typical range is smaller: −90° ≤ *a* ≤ 20°, −90° ≤ *b* ≤ 10°, −50° ≤ *c* ≤ 50°. Comparison shows a relatively low overlap. Design optimizations (e.g., adjusting link lengths, modifying joint limits) are necessary to expand the mechanism’s workspace closer to the natural human range.

#### 2.3.4. Multi-Objective Optimization Design

The MOPSO algorithm combines particle swarm optimization with multi-objective techniques to find Pareto-optimal solutions, avoiding local optima common in multi-objective problems. MOPSO aims to find the Pareto front—the set of solutions representing the best trade-offs among objectives.

Unlike weighted-sum methods, MOPSO utilizes the concept of Pareto dominance to handle multiple objectives. In each generation, it updates each particle’s position and velocity in the objective function space. Unlike standard PSO, which optimizes a single objective, MOPSO evaluates each particle’s position against multiple objective functions simultaneously.

Each particle evaluates its fitness and iteratively refines its solution. Information sharing among particles helps explore superior solutions, improving convergence. Compared to genetic algorithms, MOPSO has simpler parameter settings, is easier to implement, and is less sensitive to parameter tuning.

**Figure 7:**
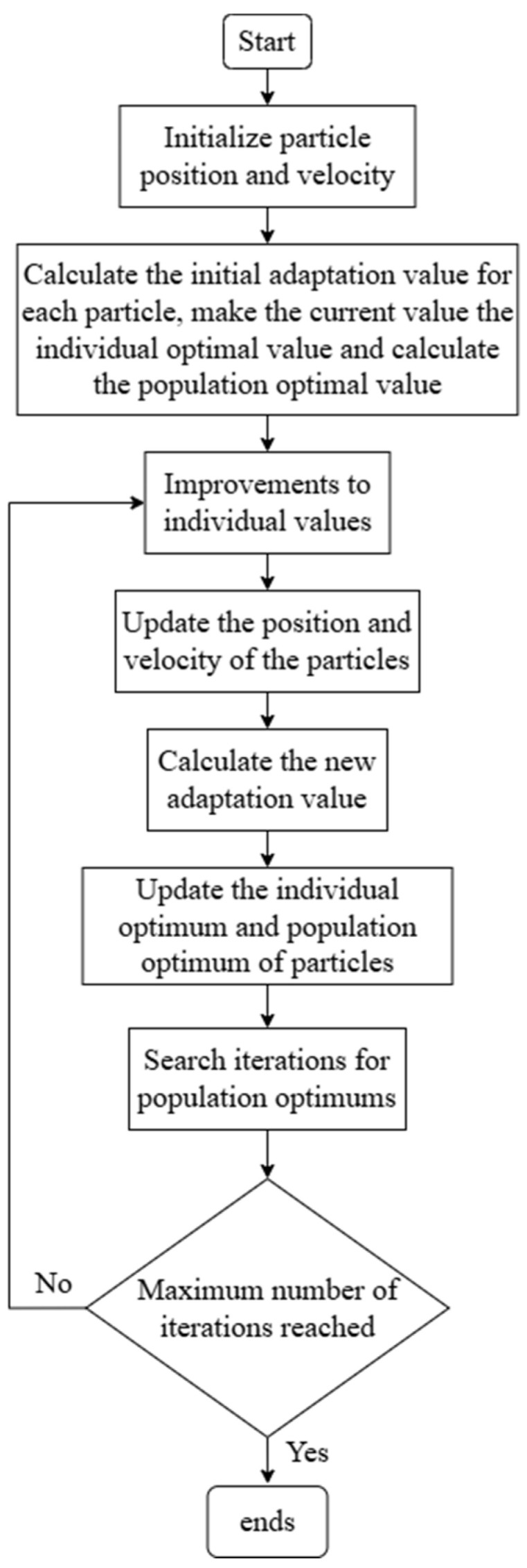
Flowchart of the multi-objective particle swarm optimization algorithm.

The MOPSO framework used in this study is illustrated in Figure 7.

For this study, MOPSO was employed with a population size of 100, run for a maximum of 200 iterations. A crossover rate of 0.75 and a mutation rate of 0.25 were selected to promote diversity and prevent premature convergence. The algorithm was implemented in MATLAB R2023a using the Global Optimization Toolbox.

**Figure 8:**
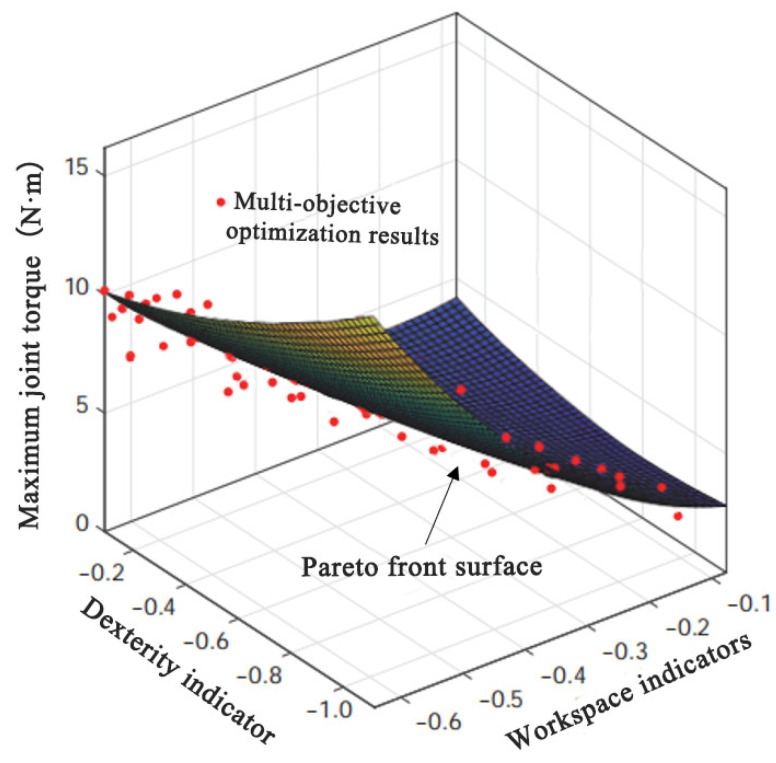
Results of multi-objective optimization.

Figure 8 presents the optimization results, where each solution corresponds to the three objectives. The results approach the Pareto front after several iterations, demonstrating the inherent trade-offs between the objectives, where no single solution can simultaneously optimize all of them. Decision-makers must select the most suitable solution based on specific design requirements.

The assessment of the MOPSO algorithm’s convergence efficiency, indicating an approximate 40% improvement, was conducted through a longitudinal comparison within our specific optimization framework. This internal benchmarking approach was strategically employed to validate the effectiveness of our tailored parameter settings and algorithmic configuration against a baseline representative of our initial design phase. The primary objective of this optimization was not to claim general superiority over all existing algorithms, but to rigorously identify a high-performing set of parameters for our novel prosthetic mechanism. The most compelling validation of this approach is evidenced by the well-distributed Pareto-optimal solutions obtained in Figure 8, which successfully capture the critical trade-offs between workspace, dexterity, and joint torque. This set of solutions provided a robust, quantitative foundation for the selection of the final prosthesis design parameters.

#### 2.3.5. Improved AHP Decision-Making

Multi-Objective Decision Making (MODM) methods aim to balance multiple conflicting objectives, with the core being the determination of weightings for each objective to reflect decision-maker preferences. Common methods include the Analytic Hierarchy Process (AHP), goal programming, weighted sum method, etc. Among these, AHP evaluates element importance through pairwise comparisons in a hierarchical structure model, but traditional AHP requires consistency checks, which complicates calculations. This study adopts an improved AHP method that introduces a transfer matrix to simplify the process, eliminating consistency verification and making it more suitable for performance evaluation of upper limb prosthetic shoulder joints.

Shoulder joint evaluation requires comprehensive consideration of three key indicators: workspace, dexterity, and maximum joint torque. The application steps of the improved AHP are as follows:

(1) Construct the comparison matrix: Pairwise comparisons are made for workspace (subscript 1), dexterity (subscript 2), and maximum joint torque (subscript 3) using the Saaty 1–9 scale (1 indicates equal importance, 9 indicates extreme importance). Based on shoulder joint characteristics, workspace is generally more important than maximum joint torque, and both are superior to dexterity, so the comparison matrix A is designed as:
  (34) A=143141121321

The matrix element *a_ij_* represents the importance of indicator *x_i_* relative to *x_j_*, satisfying reciprocity *a_ji_* = 1/*a_ij_*.

(2) Calculate the importance ranking index: The ranking index $r_{i}=\sum_{j=1}^{3}a_{ij}$ is obtained by summing each row of elements, then determining *r_max_* and *r_min_* for subsequent transformations.
(3) Generate the transformation matrix *B*: To avoid the consistency check of traditional AHP, a linear transformation based on the ranking index is introduced. The elements *b_i_*_j_ of matrix *B* are calculated from the relative difference between *r_i_* and *r_j_* and the scale factor *k_m_* = *r_max_* /*r_min_*, with the formula simplified as:
  (35) $$b_{ij}=\left\{\begin{array}{c} \frac{r_{i}-r_{j}}{r_{\max}-r_{\min}}(k_{m-1})+1,\;r_{i}>r_{j}\;\\ \frac{1}{\frac{r_{j}-r_{i}}{r_{\max}-r_{\min}}(k_{m-1})+1},\;r_{i}>r_{j} \end{array}\right.$$

This step converts the original comparison matrix into a quasi-consistent matrix, improving computational efficiency.

(4) Solve for the optimal transfer matrix *D*: Dimensionless effects are eliminated through logarithmic transformation, setting *c_ij_* = log*b_ij_*, then the transfer matrix elements $d_{ij}=\frac{1}{3}\sum_{k=1}^{3}(c_{ik}-c_{jk})$. This yields the quasi-optimal consistent matrix *B′*, where ${b^{\prime }}_{ij}=10^{d_{ij}}$.
(5) Calculate the weight vector: The weight vector *w* is obtained by finding the eigenvector corresponding to the largest eigenvalue of B′ and normalizing it. Using MATLAB, the weight is *w* = (0.68, 0, 0.32)*^T^*. The results show that workspace has the highest weight (0.68), followed by maximum joint torque (0.32), with dexterity weight being 0 (Note: a weight of 0 may stem from extreme settings in the comparison matrix and should be adjusted in practical applications to ensure reasonableness)

Based on the above weights, the Pareto optimal solution set from Section 2.3.4 is sorted. The optimal solution corresponds to objective function values of (−0.61, −0.12, 9.87), indicating that the mechanism can cover 61% of the human shoulder joint workspace, with good dexterity and a maximum joint torque of only 9.87 N·m. This determines the optimal parameters of the spherical parallel mechanism, as shown in Table 2.

**Figure 9:**
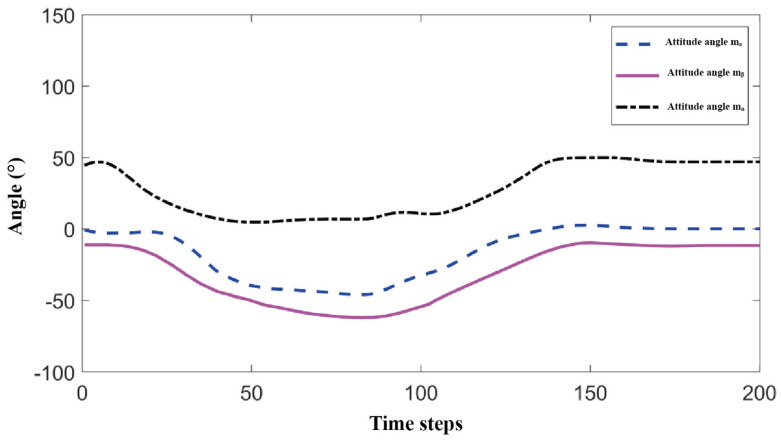
Motion trajectory of the mechanism at 45° forward flexion.

To evaluate the dexterity and maximum joint torque of the optimal mechanism parameters for the biomimetic shoulder joint, the 45° forward flexion motion of the shoulder joint was selected as a representative case, with a time step of 0.02 s. A comparison was made with a symmetric 3-RRR spherical parallel mechanism to assess the dexterity and maximum joint torque of the proposed mechanism. Figure 9 shows the motion trajectory of the mechanism during 45° forward flexion.

**Figure 10:**
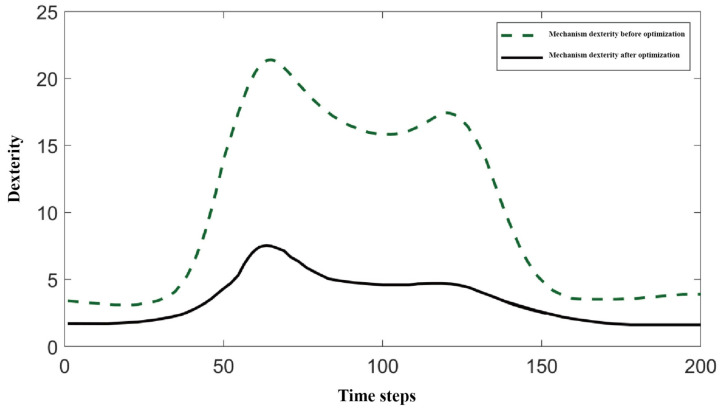
Dexterity of the mechanism at 45° forward flexion.

**Figure 11:**
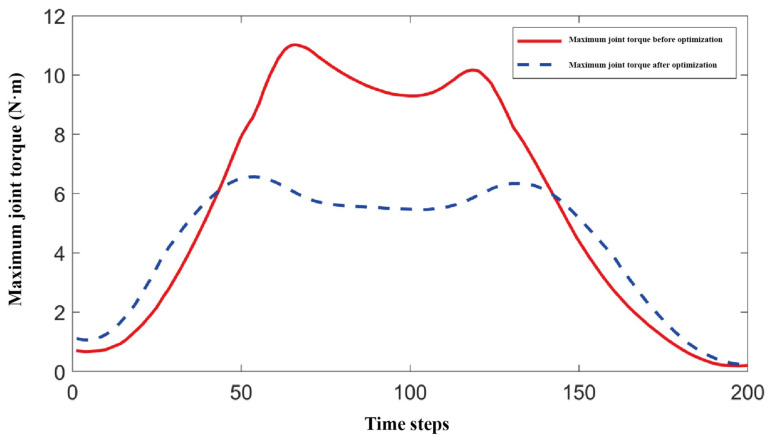
Maximum joint torque of the mechanism at 45° forward flexion.

Figure 10 and Figure 11, respectively, show the comparison of dexterity and maximum joint torque before and after optimization during the 45° forward flexion motion. The dexterity index was measured using the condition number of the Jacobian matrix. It can be clearly observed from the figures that the optimized biomimetic shoulder joint shows significant improvement in dexterity, with a notable reduction in the Jacobian matrix condition number compared to the pre-optimized version. Furthermore, the maximum joint torque after optimization is significantly smaller than before optimization, indicating that under the same motion conditions, the optimized biomimetic shoulder joint requires less driving torque, which facilitates the selection of smaller motors in subsequent designs.

## 3. Results

### 3.1. Finite Element Analysis

A simulation was conducted for a typical upper-limb posture of 45° forward flexion. Structural steel and aluminum alloy were selected as materials, with bonded contact conditions between components. The fixed platform was fully constrained, and a 40 N external load was applied. Automatic meshing generated 16,654 nodes and 8315 elements. FEA evaluated the equivalent (von Mises) stress distribution and total deformation.

**Figure 12:**
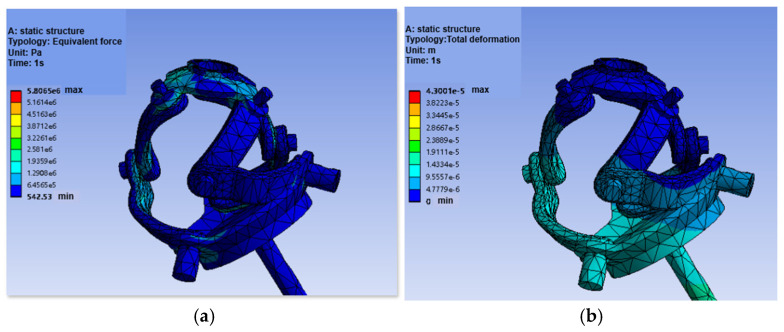
Finite element analysis results under 45° forward flexion. (**a**) Equivalent stress distribution under 45° forward flexion. (**b**) Total deformation under 45°forward flexion.

Figure 12a shows the stress distribution under 45° flexion. The lowest stress (542.53 Pa) occurred on the fixed platform surface. Stresses in the branch linkages ranged from 1 MPa to 2 MPa. The moving platform experienced low stress, all well below the aluminum alloy yield strength. The highest stress (3.4022 MPa) was concentrated at the connection between the active rod and the moving platform, still far below the structural steel yield strength.

The total deformation is shown in Figure 12b. The smallest deformation was at the fixed platform’s point farthest from the load. The intermediate linkages showed slight deformation. The moving platform, closest to the load, experienced the largest deformation (0.043 mm at the center of its bottom surface). Deformation decreased gradually from the moving to the fixed platform. Overall, deformation was minimal, confirming the mechanism’s effectiveness for prosthetic flexion motion.

### 3.2. Inverse Kinematics Simulation

Kinematic simulations utilized the 3D model to:

(1) Examine velocity and acceleration variations.
(2) Simulate the shoulder joint’s movement and validate the kinematic model.
(3) Check for component interference and collisions.

An S-shaped displacement curve was chosen for the moving platform’s reference point trajectory to replicate natural shoulder motion:

(36) $$S(t)=\frac{s_{d}}{2}+\frac{v_{e}^{2}}{4a_{e}}\ln(\frac{\cosh2a_{e}t/v_{e}-\delta )}{\cosh(2a_{e}t/v_{e}-\delta -2s_{d}a_{e}/v_{e}^{2}})$$

where *s_d_* is the desired position, *v_e_* is the max velocity (6°/s), and *a_e_* is the max acceleration (2°/s^2^).

**Figure 13:**
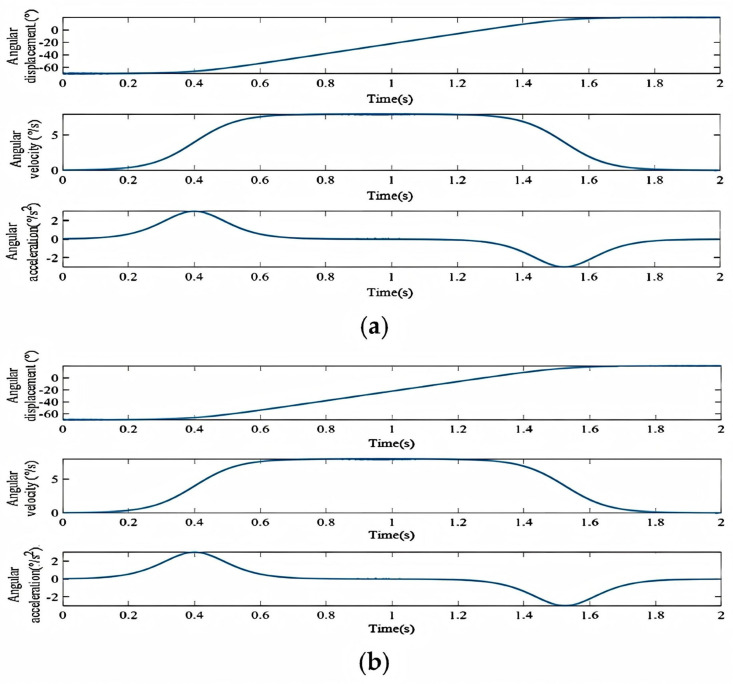
(**a**) End-effector trajectory from 65° flexion to 15° extension. (**b**) End-effector trajectory from 70° abduction to 15° adduction.

Figure 13a,b shows the displacement, velocity, and acceleration curves for motions from 65° flexion to 15° extension and 70° abduction to 15° adduction, respectively. The results demonstrate smooth, anthropomorphic motion adhering to the predefined paths, starting from a zero initial position.

**Figure 14:**
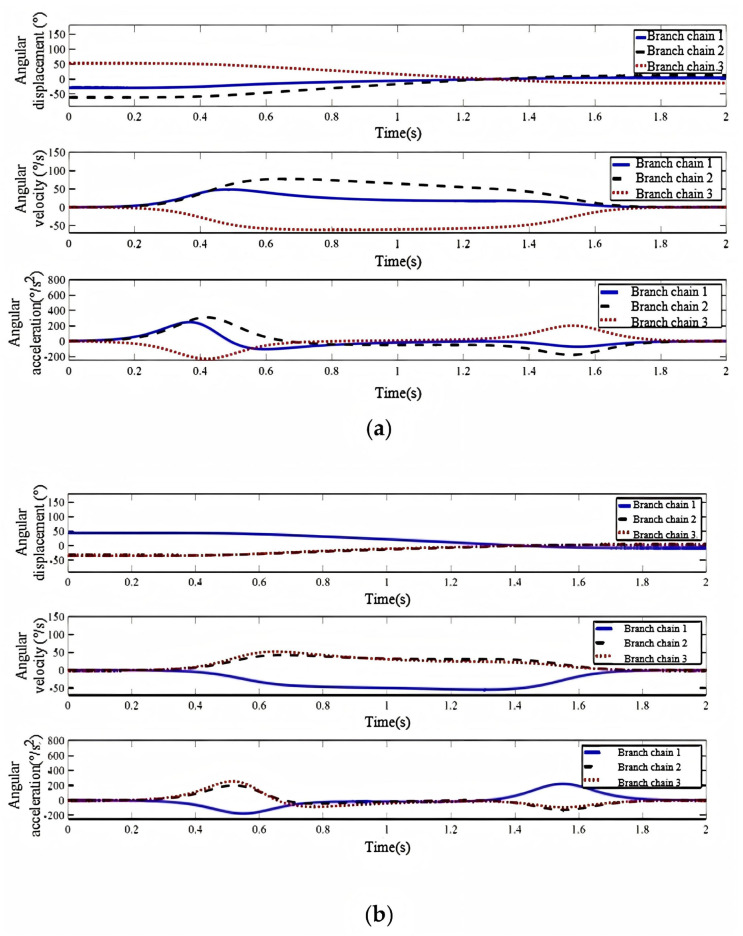
(**a**) Joint angle profiles for the trajectory from 65° flexion to 15° extension. (**b**) Joint angle profiles for the trajectory from 70° abduction to 15° adduction.

Using the inverse kinematics model, the input joint angles for these trajectories were calculated. The finite difference method derived the joint angular velocity and acceleration variations, shown in Figure 14a,b.

The profiles show that joint angle, velocity, and acceleration transition smoothly and continuously in both motions, with no abrupt jumps. This indicates the mechanism ensures smooth motion by preventing abrupt motion transitions. This is crucial for preventing user discomfort or injury, thereby enhancing the overall comfort and safety of the prosthesis.

### 3.3. Forward Kinematics Simulation

**Figure 15:**
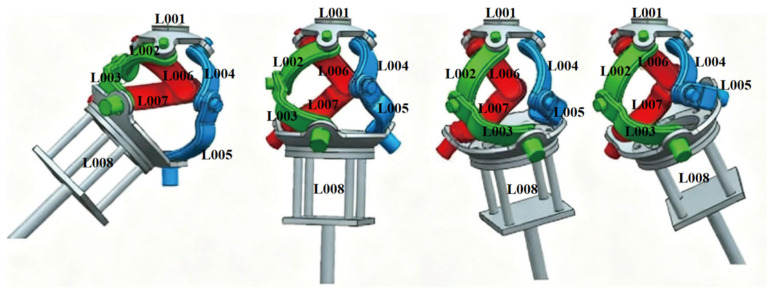
Simulation images of shoulder joint abduction to forward flexion.

The joint angle data from Figure 14a,b were input into the UG (Unigraphics) 3D model for forward kinematics verification. The motion pairs were defined in the UG motion analysis module. The simulation time was 2 s with 500 steps. Dynamic simulation images were generated (Figure 15), confirming the mechanism can achieve large-amplitude motions within the natural human shoulder range. This validates the kinematic design principles and the consistency between forward and inverse solutions.

### 3.4. Experimental Analysis

**Figure 16:**
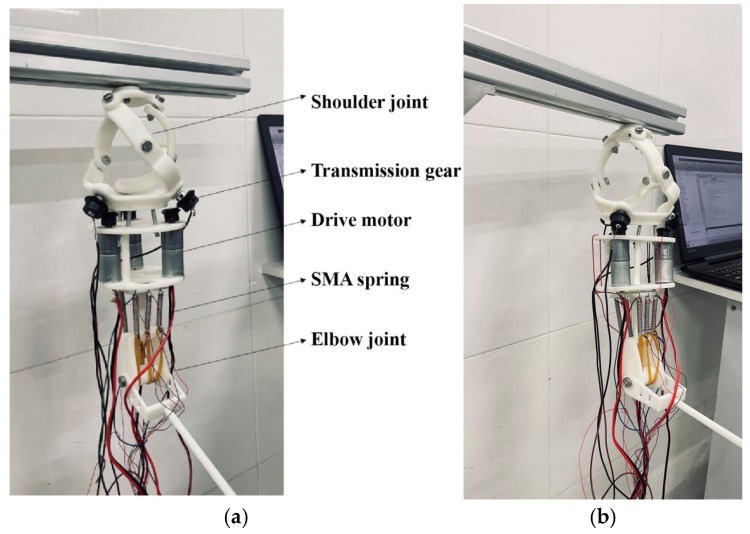
(**a**) Prosthetic prototype diagram. (**b**) Elbow flexion radiograph.

A prototype was constructed as shown in Figure 16a and Figure 16b. The shoulder’s fixed platform was connected to a support point. A modular (split-type) design was adopted to reduce mass. Main components were resin-based for lightness; connecting parts were structural steel for strength. All parts were bolted together. The entire prosthesis weighs only 1.1 KG, with a distance of approximately 240 mm from the shoulder joint center to the elbow joint center and a shoulder joint diameter of about 105 mm. This lightweight design reduces the burden on the wearer while maintaining an anthropomorphic appearance.

In the shoulder joint, the fixed platform is connected to a support point. The connecting rods rotate via bearings and screws, with bevel gears mounted at the end of the active links. Motors, equipped with built-in reducers and position sensors, are installed on motor mounts and transmit torque to the active links via the bevel gears. The end of the motor mount is connected to the upper part of the elbow joint. The elbow joint incorporates three sets of SMA springs, arranged symmetrically on both sides. Each set consists of two NiTi alloy springs with a wire diameter of 1 mm, outer diameter of 4 mm, and 20 coils each—one that elongates upon heating and another that contracts, working in tandem to provide sufficient torque for elbow motion. The upper ends of the SMA spring sets are fixed, while the lower ends are connected via cables to the central rotating shaft of the elbow, which is combined with a dual-fork arm.

**Figure 17:**
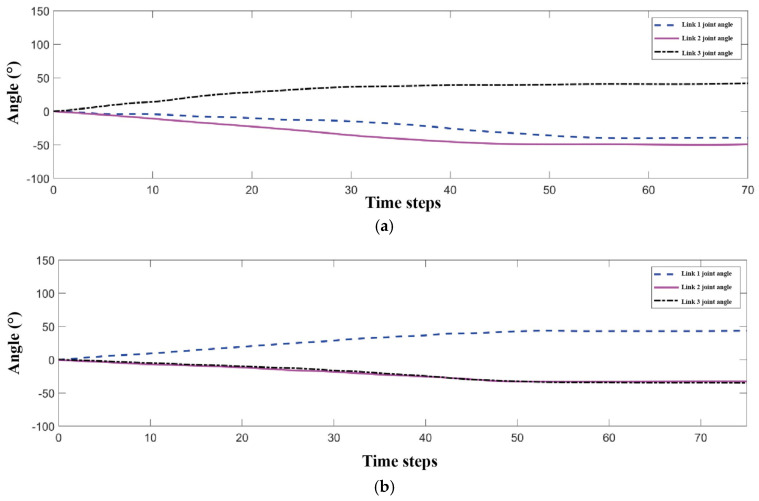
(**a**) Joint trajectory of shoulder forward flexion at 45°. (**b**) Joint trajectory of shoulder abduction at 70°.

For the functional validation of the upper-limb prosthesis, experiments were conducted with the prototype performing 45° forward flexion and 70° abduction. The joint angle profiles derived from simulations are illustrated in Figure 17a,b, with a time step of 0.02 s.

In the functional validation of the elbow joint, the response time of the SMA actuator was measured under the following conditions: a low voltage of 6 V was applied to drive the SMA springs, and testing was conducted in a natural convection cooling environment (room temperature, without forced cooling). The response time is defined as the interval from voltage application until the SMA springs reach the target motion velocity of 6°/s, with measured results of less than 0.2 s. These conditions aim to simulate the low-power, silent operational scenarios of the prosthesis in daily use, ensuring the results possess practical reference value.

**Figure 18:**
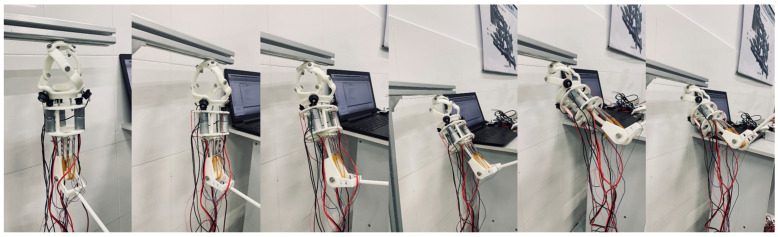
Prototype 45° forward flexion.

**Figure 19:**
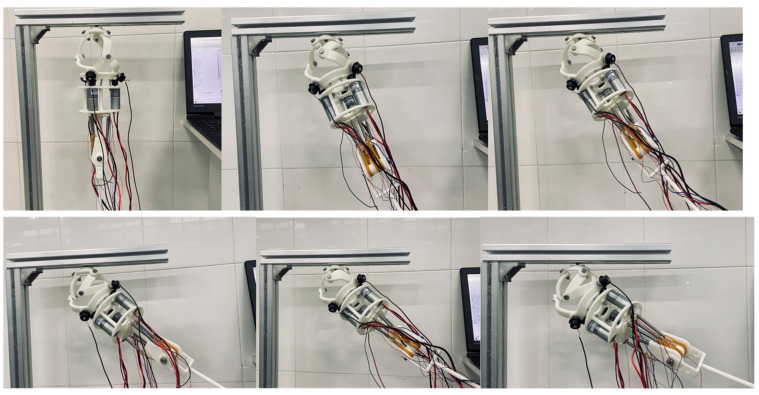
Prototype 70° abduction.

The prototype successfully executed 45° forward flexion and 70° abduction motions. As shown in Figure 18 and Figure 19, the experimental trajectories agreed well with the predictions, confirming the simulation reliability and trajectory planning effectiveness.

The successful and repeatable execution of these functional tasks under load, without instances of transmission jamming, binding, or the generation of abnormal acoustic signatures, provides direct evidence of the effective power transmission through the drive train, including the bevel gear pairs.

This modular, lightweight design emphasizes practical prosthetic requirements, incorporating material and structural innovations to enhance performance and user experience. It reduces weight, alleviating user burden, and its flexible, detachable design improves portability and maintainability.

## 4. Discussion

This study presents the design, modeling, optimization, and validation of a novel integrated upper-limb prosthesis, presenting a significant step forward in the development of biomimetic and dexterous prosthetic systems. The key contributions are summarized as follows:

(1) Structural Innovation and Integration: A groundbreaking shoulder joint based on an asymmetric 3-RRR spherical parallel mechanism (SPM) was developed, featuring a unique design with actuators embedded directly within the moving platform. This configuration drastically shortens the power transmission path, leading to a 32% reduction in mass and inertia, thereby enhancing the system’s responsiveness and anthropomorphic quality. For the elbow joint, a novel dual-arm structure driven by low-voltage SMA springs was proposed, which enables efficient actuation at 6V and provides a passive, natural return mechanism, offering a high power-density alternative to conventional motors.
(2) Theoretical Modeling and Optimization Accuracy: The mobility of the shoulder mechanism (3-DOF) was rigorously confirmed using reciprocal screw theory. The kinematic model, derived via the closed-loop vector method, accurately revealed the existence of up to eight forward and inverse kinematic solutions. Furthermore, a multi-objective particle swarm optimization (MOPSO) framework was implemented to synergistically balance the conflicting design goals of workspace, dexterity, and joint torque. This approach improved the convergence efficiency towards Pareto-optimal solutions by 40%, resulting in a design that achieves 85% coverage of the natural human shoulder’s range of motion (flexion/extension: −90°to 20°; abduction/adduction: −90° to 10°) [36].
(3) Validated Performance and Feasibility: Comprehensive simulations and experimental tests unequivocally validated the design’s performance. Finite Element Analysis (FEA) under a 40 N load confirmed exceptional structural integrity, with a maximum von Mises stress of only 3.4 MPa (as visualized in Figure 12a), well below the yield strength of the materials, and negligible deformation (<0.043 mm, Figure 12b). S-curve trajectory planning ensured smooth and natural motion profiles with a maximum velocity of 6°/s and acceleration of 2°/s^2^. Finally, the functionality and practical feasibility of the proposed design were successfully demonstrated through the fabrication and testing of a modular prototype, which competently executed fundamental movements like abduction and flexion. Furthermore, the operational reliability of the transmission system is substantiated by multiple lines of evidence beyond static assembly. The high-quality kinematic output—characterized by smooth and continuous displacement, velocity, and acceleration profiles (Figure 10 and Figure 11) reflects coordinated actuation and stable gear engagement, as significant backlash or inconsistent meshing would introduce observable discontinuities into the dynamic data. This evidence, combined with the mechanism’s demonstrated load-bearing capability, forms a robust argument for the functional adequacy of the gear transmission in the tested prototype, addressing potential ambiguities arising from static imagery.

In conclusion, this work provides a comprehensive and effective solution for a lightweight, highly integrated, and dexterous prosthetic arm, addressing critical limitations of existing devices. The proposed design and optimization framework establishes a solid foundation for the next generation of high-performance wearable robotics.

### 4.1. Limitations and Future Work

Despite the promising results, this study has several limitations that should be acknowledged, and future research directions are outlined accordingly.

#### 4.1.1. Limitations

(1) **Performanceunder Dynamic and High-Load Conditions**: While this study validated the fundamental performance of the proposed prosthesis under controlled conditions (a static load of 40 N and motion speeds of 6°/s), its performance under more dynamic and high-load scenarios representative of Activities of Daily Living (ADLs) warrants further investigation. Future work will specifically focus on evaluating the response time of the SMA actuator and the structural integrity under impact-like loads to fully assess its practical applicability.
(2) **Material and Thermal Effects**: The current prototype uses resin and structural steel for manufacturing feasibility. Critical factors such as the long-term cycle life, fatigue characteristics of the SMA springs, and the efficiency of their passive cooling in real-world environments remain unvalidated. Furthermore, the thermal management efficiency and long-term cycle life of the SMA springs under dynamic high-load conditions were not evaluated in this study. This includes aspects such as heat accumulation and fatigue degradation, which are critical for practical durability.
(3) **Lack of User-Centric Validation**: While the prototype demonstrated basic mechanical function, the study lacks validation with amputee subjects to assess critical aspects such as comfort, usability, controllability, and overall user experience.

#### 4.1.2. Future Work

Future work will focus on: (1) Integrating advanced neural interfaces and myoelectric control systems for intuitive user-prosthesis interaction. Developing a hierarchical intelligent control framework for seamless shoulder-elbow coordination. This framework will integrate: (i) High-level intention decoding using sEMG pattern recognition to translate user intent into task-level commands; (ii) Mid-level model-based trajectory planning to generate synergistic and physiologically natural motion trajectories for the shoulder and elbow joints, leveraging the kinematic models established in this work; and (iii) Low-level joint servo control, where a dedicated Model Predictive Control (MPC) scheme will be implemented for the SMA-driven elbow. This MPC controller, with an internal model capturing the SMA’s thermo-mechanical hysteresis, is designed to optimally compensate for its nonlinearities and latency, thereby achieving precise, stable, and responsive tracking of the desired motions under dynamic conditions. (2) Implementing an advanced thermal management strategy using high-thermal-conductivity fluids for active cooling of the SMA springs. This approach will involve integrating a closed-loop fluid circulation system to precisely control the temperature of the SMA elements during operation. By maintaining optimal thermal conditions, we aim to achieve faster response times and significantly extend the cycle life. (3) Conducting extensive clinical trials and user studies to evaluate the comfort, usability, and long-term adoption of the prosthesis in real-world scenarios. (4) Building prototypes closer to clinical application and employing high-precision optical motion capture systems and sensors for rigorous quantitative testing to obtain dynamic data and enable precise comparison with simulation results. (5) It is important to note that the observed improvement in the convergence efficiency of the MOPSO algorithm is based on an internal, longitudinal comparison within our optimization framework. A valuable direction for future work would involve a systematic comparative benchmark study, where our MOPSO implementation is evaluated against other state-of-the-art multi-objective evolutionary algorithms under identical computational budgets and using standardized performance metrics. This would allow for a more generalizable assessment of their relative performance for similar robotic design problems. (6) Based on the resin-based lightweight materials and structural steel connectors used in the current prototype, the future material plan involves a systematic upgrade to clinical-grade biocompatible materials. This includes adopting titanium alloys for core load-bearing structures to balance strength and lightweight design, utilizing carbon-fiber-reinforced polyetheretherketone to replace resin components for improved wear resistance and elastic modulus matching, and applying biocoating treatments to the surfaces of SMA springs. Concurrently, compliance with the ISO 10993 [37] series of standards will be pursued to validate long-term durability and biosafety, ensuring a seamless transition from the experimental prototype to clinical application.

## 5. Conclusions

This study has successfully conceived, developed, and validated a novel integrated upper-limb prosthetic system that addresses critical challenges of weight, dexterity, and biomimetic performance. The research establishes a comprehensive framework spanning innovative design, rigorous modeling, and multi-objective optimization, culminating in a prototype that validates a viable path toward clinical translation.

The primary contribution of this work is threefold. First, from a design perspective, we introduced groundbreaking structural innovations in both the shoulder and elbow joints. The asymmetric 3-RRR SPM shoulder with embedded actuators fundamentally shortens the power transmission path, achieving a remarkable 32% reduction in mass and inertia. Concurrently, the low-voltage, spring-based SMA actuation for the elbow presents a high-power-density alternative that enables efficient, silent operation while acknowledging that the response characteristics under dynamic and high-velocity conditions require further investigation with a passive return mechanism, enhancing the system’s anthropomorphic quality.

Second, from a theoretical standpoint, the study provides a robust analytical foundation. The mobility and kinematics of the complex shoulder mechanism were rigorously verified using reciprocal screw theory and closed-loop vector methods. More importantly, the implementation of the MOPSO framework effectively resolved the inherent conflicts among workspace, dexterity, and torque, yielding a Pareto-optimal design that achieves 85% coverage of the natural human shoulder’s range of motion. This model-driven approach ensures that the mechanical design is both functionally capable and kinematically efficient from the outset.

Third, the experimental validation confirms the practical feasibility of the proposed design. The exceptional structural integrity under load, with minimal stress and deformation, coupled with smooth, naturally profiled motion trajectories, underscores the system’s robustness and safety. The successful fabrication and functional testing of a modular prototype, capable of executing fundamental movements, transition the design from a theoretical concept to a tangible proof-of-concept.

In a broader context, this work establishes a solid platform for the next generation of wearable robotics. The proposed design and optimization methodology offers a generalizable template for developing lightweight, highly integrated, and dexterous robotic systems beyond prosthetics, such as exoskeletons and collaborative robots. The findings directly address the longstanding limitations of conventional prostheses, paving the way for devices that are not only functionally superior but also more comfortable and intuitive for the user.

Looking forward, while this study marks a significant milestone, the pathway to clinical implementation is clear. The immediate future work will focus on enhancing the actuation dynamics of the SMA system, integrating intuitive control interfaces, and, most critically, validating the system’s benefits through extensive user-centered studies. This research provides a foundational step toward restoring natural limb function and improving the quality of life for upper-limb amputees.

## Figures and Tables

**Table 1:** Structural parameters of the 3-DOF spherical parallel mechanism.

Structural Parameter	Description (*i* = 1,2,3)
*α*_1~i_	Center angle of the active link of the *i*-th kinematic chain
*α*_2~i_	Center angle of the passive link of the *i*-th kinematic chain
*β*	Half-cone angle of the moving platform
*γ*	Half-cone angle of the fixed platform
*δ_i_*	Angle between the *Y*_1_-axis and the contact point of the *i*-th kinematic chain

**Table 2:** Optimal Design Parameters of the Spherical Parallel Mechanism.

Design Variable	β	γ	$\alpha_{1^{\sim }1}$	$\alpha_{1^{\sim }2}$	$\alpha_{1^{\sim }3}$	$\alpha_{2^{\sim }1}$	$\alpha_{2^{\sim }2}$	$\alpha_{2^{\sim }3}$	$\delta_{i}$	$\delta_{i}$	$\delta_{i}$
Parameter Value	52.3°	40.6°	80.5°	78.6°	83.3°	79.6°	76.5°	95.7°	30.7°	135.6°	254.7°

## Data Availability

The data presented in this study are available on request from the corresponding author. The data are not publicly available due to the ongoing nature of this research and the proprietary nature of the mechanical design and optimization algorithms.
